# Liraglutide Decreases Liver Fat Content and Serum Fibroblast Growth Factor 21 Levels in Newly Diagnosed Overweight Patients with Type 2 Diabetes and Nonalcoholic Fatty Liver Disease

**DOI:** 10.1155/2021/3715026

**Published:** 2021-10-08

**Authors:** Xinyue Li, Xiaojuan Wu, Yumei Jia, Jing Fu, Lin Zhang, Tao Jiang, Jia Liu, Guang Wang

**Affiliations:** ^1^Department of Radiology, Beijing Chao Yang Hospital, Capital Medical University, No. 8, Gong ti South Road, Chao Yang District, Beijing 100020, China; ^2^Department of Endocrinology, Beijing Chao Yang Hospital, Capital Medical University, No. 8, Gong ti South Road, Chao Yang District, Beijing 100020, China

## Abstract

**Purposes:**

In this study, we aimed to verify plasma fibroblast growth factor 21 (FGF21) elevation in newly diagnosed overweight patients with type 2 diabetes mellitus (T2DM) and nonalcoholic fatty liver disease (NAFLD) and to evaluate the effectiveness of liraglutide on reducing liver fat content and serum (FGF21) levels in those patients.

**Methods:**

A 12-week, single-center, prospective study was conducted. Twenty newly diagnosed overweight patients with T2DM and NAFLD were recruited. Twenty healthy age, sex, and body mass index (BMI) matched subjects were enrolled as the control group. Enzyme-linked immunosorbent assay was used to measure serum FGF21 levels. Liver fat content was determined using the 3.0 T whole-body MRI scanner.

**Results:**

Those newly diagnosed overweight patients with T2DM and NAFLD had a BMI of 27.6 ± 0.5 kg/m^2^. They had higher levels of FGF21 (159.6 ± 35.7 vs. 124.1 ± 42.9 pg/ml, *P* < 0.001) and increased liver fat content (19.3 ± 9.4 vs. 4.5 ± 0.6%, *P* < 0.001) compared to the controls. Liraglutide treatment for 12 weeks induced a significant 4.9 kg weight loss (95% confidence interval (CI): -6.1, -3.7, *P* < 0.001), which was equivalent to a relative reduction of 6.8% (95% CI: 5.3%, 8.3%, *P* < 0.001). FGF21 levels decreased after the 12-week liraglutide treatment (159.6 ± 35.7 vs. 124.2 ± 27.8 pg/ml, *P* = 0.006). There was a positive correlation between relative changes of liver fat content and relative change of FGF21 (*r* = 0.645, *P* = 0.002). FGF21 levels significantly decreased in patients who had a significant decrease in liver fat content (≥29%) (95% CI: -262.8, -55.1, *P* = 0.006); however, there was no significant change in the patients without a significant decrease in liver fat content (<29%) (95% CI: -60.0, 54.1, *P* = 0.899).

**Conclusions:**

Liraglutide treatment reduced both liver fat content and FGF21 levels in newly diagnosed overweight patients with T2DM and NAFLD. FGF21 may be a potential biomarker for evaluating the effects of liraglutide treatment on hepatic fat and glucose metabolism.

## 1. Introduction

Nonalcoholic fatty liver disease (NAFLD) is one of the most common liver diseases around the world affecting approximately 25% of the global population [1]. NAFLD is also one of the prominent causes of chronic liver disease in patients with diabetes. It has been reported that 50% to 75% of diabetic patients suffer from NAFLD [2]. Lifestyle adjustments, including physical activity and rational diet, are the main management strategies for NAFLD, but these approaches are not effective long term. As a long-acting glucagon-like peptide-1 (GLP-1) analogue, liraglutide induces central appetite suppression, gastric emptying delay, and weight loss [3]. Therefore, liraglutide has become a promising therapeutic option for patients with nonalcoholic steatohepatitis (NASH) [4].

Fibroblast growth factor 21 (FGF21) is a protein that is synthesized in the liver, adipose tissue, and pancreas. FGF21 increases insulin sensitivity, stimulating fatty acid oxidation in the liver and glucose uptake in fat tissues [5]. Generally, circulating FGF21 is mainly derived from the liver [6, 7], and increased FGF21 levels have been observed in both obese mice [8] and patients [9]. Previous studies have also shown that circulating FGF21 levels are significantly elevated in type 2 diabetes mellitus (T2DM) patients [10–13].

Here, we explored the effects of the GLP-1 analogue liraglutide on liver fat content and serum FGF21 levels in newly diagnosed overweight patients with T2DM and NAFLD.

## 2. Methods

### 2.1. Subjects

This prospective study was performed in the Department of Endocrinology, Beijing Chao Yang Hospital of Capital Medical University between November 2018 and October 2019. We consecutively enrolled 20 newly diagnosed overweight patients complicated with T2DM and NAFLD and 20 healthy age, sex, weight, and body mass index (BMI) matched subjects as the control group. The inclusion criteria were as follows: (i) 18-65 years of age; (ii) all T2DM patients had no diabetes antibodies and were newly diagnosed within 3 months, according to the ADA diagnostic criteria, and glycated hemoglobin (HbA1c) levels were ≥6.5% (49 mmol/mol) [14]; (iii) 25 ≤ BMI < 30 kg/m^2^ [15]; and (iv) liver fat content > 5.5% [16]. Exclusion criteria were as follows: (i) alcohol consumption (>70 g/week for women or >140 g/week for men); (ii) other causes of liver disease (i.e., acute and chronic viral hepatitis, Wilson's disease, infectious liver disease, and autoimmune hepatitis); (iii) confounding concomitant medication (i.e., thiazolidinediones, vitamin E, fenofibrate, and statin); (iv) medical history of pancreatitis, pancreatic or thyroid carcinoma, type 1 diabetes mellitus, diabetic ketoacidosis, diabetic hyperosmolar coma, nephropathy, acute or chronic hepatic and renal diseases, severe anemia, acute myocardial infarction, or stoke; and (v) magnetic resonance imaging (MRI) contraindications. All enrolled participants provided informed consent, and the experimental design was approved by the Ethics Committee of Beijing Chao Yang Hospital, Capital Medical University.

### 2.2. Study Design

All participants were subjected to physical examination (height, weight, and waist circumference) and laboratory inspection. Blood sampling of subjects was conducted in the morning after overnight fasting, and the specimens were then stored at −80°C. Colorimetric enzymatic assays were applied to measure the levels of total cholesterol (TC), low-density lipoprotein cholesterol (LDL), high-density lipoprotein cholesterol (HDL), triglyceride (TG), alanine amino transferase (ALT), aspartate aminotransferase (AST), alkaline phosphatase (ALP), and *γ*-glutamyl transferase (GGT) using an autoanalyzer (Hitachi 7170). Fasting blood glucose (FBG), 2 h postprandial blood glucose (2 hPBG), fasting insulin (FINS), and HbA1c levels were determined in the central chemistry laboratory of Beijing Chao Yang Hospital, Capital Medical University. The plasma concentration of FGF21 was quantified using enzyme-linked immunosorbent assay (ELISA) according to the manufacturer's protocols (Millipore, USA). The intra- and interassay coefficients of variation were 3.9% and 10.9%, respectively. BMI was calculated as weight (kg)/height (m^2^). The homeostasis model assessment of insulin resistance (HOMA-IR) index was calculated according to the formula: HOMA‐IR = FINS(*µ*IU/mL) × FBG(mmol/L)/22.5 [17].

To improve gastrointestinal tolerability, all enrolled T2DM patients first received a fortnight dose adjustment of liraglutide: the initial dose was set as 0.6 mg/d, and the dose increase per week was 0.6 mg. The final dose of 1.8 mg was employed in the following study. All patients at baseline had accepted dietary advice from a dietitian and physical advice from a nurse according to the Guidelines for Prevention and Treatment of Type 2 Diabetes in China (2017 Edition). Overweight patients were required to reduce calorie intake on an individual basis. All patients were recommended to control the fat intake to be less than 30% and take foods rich in monounsaturated fat. All patients were advised to exercise for at least 3 days per week for a total of at least 150 minutes. And all patients received no antidiabetic regimen at baseline. After liraglutide treatment for 12 weeks, all patients were subjected to follow-up evaluation, including physical examination and blood laboratory testing. Levels of TC, LDL, HDL, TG, FBG, FINS, HbA1c, FGF21, and 2 hPBG were detected. HOMA-IR was also calculated. All above blood measurements were performed at the basal state. The safety outcomes included gastrointestinal reactions (i.e., poor appetite, nausea, and diarrhea).

### 2.3. MRI Examination

All subjects received upper-abdominal MRI examinations to accurately measure liver fat content before and after the 12-week liraglutide treatment. Each subject underwent an upper-abdominal MRI examination with a 3-Tesla whole-body human MRI scanner (MAGNETOM Prisma System; Siemens Medical Solutions, Erlangen, Germany) while in the supine position. Subjects were instructed to follow a 10 to 12 h overnight fast before imaging. The scanning protocol comprised an initial set of localizer images and then a T1 volumetric interpolated breath-hold examination (VIBE) Dixon sequence. The scans covered all upper abdominal organs, including liver and pancreas. The imaging parameters were as follows: TE1 1.23 ms, TE2 2.46 ms, TR 3.97 ms, 9° flip angle, Bandwidth1 1040 Hz/Px, Bandwidth2 1040 Hz/Px, and a slice thickness of 3.0 mm. All subjects were carefully instructed to hold their breath during end expiration to ensure consistency.

### 2.4. MR Image Postprocessing and Analysis

A Siemens Syngo.via image processing workstation was used for analysis. The in-phase and fat-phase raw data were sent to the workstation to acquire the fat-fraction map image. To estimate hepatic proton density fat fraction (PDFF), the signal intensities from regions of interest (ROIs) in the liver were calculated in a fat-fraction map image with the MITK 3M3 software (downloadable at http://http://www.mitk.org/). ROIs covering the whole liver, ensuring devoid of large vessels, ducts, organ boundaries, focal hepatic lesions, and imaging artifacts, were placed manually by an experienced radiologist. The same radiologist performed both image processing and data reading for each group, and a blinded senior radiologist supervised the quality of image and measurement data.

### 2.5. Statistical Analysis

Data were analyzed using SPSS 22.0 (SPSS Inc., Chicago, IL, USA). Continuous data are expressed as mean ± standard deviation (S.D.). Noncontinuous variables are presented as medians (25^th^ and 75^th^ quartiles). Differences between healthy controls and overweight patients were compared using the Student *t*-test or nonparametric Mann-Whitney *U* test. Changes in parameters from baseline within groups were evaluated using a two-tailed paired *t*-test or rank sum test. Simple linear regression analyses were conducted by setting FGF21 baseline levels as the dependent variable. The baseline age, weight, BMI, waist circumference, and all biochemical parameters were indicated as independent variables. The association between relative change of FGF21 and that of liver fat content was also evaluated using simple linear regression analysis. Statistical significance was set as *P* < 0.05.

## 3. Results

### 3.1. Baseline Characteristics

As shown in Table 1, there were no differences in age, sex, and BMI between the two groups (*P* > 0.05). However, the T2DM patients presented higher waist circumference, FBG, 2 hPBG, FINS, HOMA-IR, HbA1c, TCH, TG, ALT, AST, and GGT and lower HDL levels than the control subjects (*P* < 0.05). Patients with both T2DM and NAFLD had higher levels of serum FGF21 (159.6 ± 35.7 vs. 124.1 ± 42.9 pg/ml, *P* < 0.001) and liver fat content (19.3 ± 9.4 vs. 4.5 ± 0.6%, *P* < 0.001) than the controls.

For all subjects at baseline, simple linear regression analysis revealed that the serum FGF21 levels were positively correlated with a series of glucose and lipid metabolic indexes (FBG (*r* = 0.575, *P* < 0.001), 2 hPBG (*r* = 0.515, *P* = 0.001), FINS (*r* = 0.808, *P* < 0.001), HOMA-IR (*r* = 0.788, *P* < 0.001), HbA1c (*r* = 0.857, *P* < 0.001), and TCH (*r* = 0.389, *P* = 0.014)), liver enzyme content (ALT (*r* = 0.649, *P* < 0.001), AST (*r* = 0.591, *P* < 0.001), and GGT (*r* = 0.537, *P* < 0.001)), and liver fat content (*r* = 0.717, *P* < 0.001) (Table 2). However, serum FGF21 levels were negatively correlated with blood HDL levels (*r* = −0.383, *P* = 0.016).

### 3.2. Effects of Liraglutide on Physical and Biochemical Parameters

As shown in Table 3, liraglutide treatment for 12 weeks resulted in a 4.9 kg absolute weight loss (95% confidence interval (CI): -6.1, -3.7, *P* < 0.001), equivalent to a 6.8% reduction (95% CI: 5.3%, 8.3%, *P* < 0.001). There were also significant decreases in BMI (27.6 ± 0.4 vs. 25.7 ± 0.7 kg/m^2^, *P* < 0.001) and waist circumference (91.0 ± 4.1 vs. 85.0 ± 2.7 cm, *P* < 0.001).

Compared to baseline, the levels of FBG (9.9 ± 2.8 vs. 6.3 ± 1.1 mmol/L, *P* < 0.001), 2 hPBG (16.5 ± 5.5 vs. 7.8 ± 1.6 mmol/L, *P* < 0.001), and HbA1c (10.0 ± 2.0 vs. 6.2 ± 0.6%, *P* < 0.001) were decreased after liraglutide treatment. We also found a reduction of HOMA-IR (6.7 ± 2.9 vs. 4.9 ± 2.1, *P* < 0.05), which indicates an attenuation of insulin resistance. Liraglutide also decreased the liver enzyme content (ALT: 56.4 ± 35.4 vs. 32.4 ± 23.1 U/L, *P* < 0.001; AST: 38.3 ± 18.7 vs. 23.0 ± 7.9 U/L, *P* < 0.001; and GGT: 79.1 ± 69.1 vs. 46.8 ± 26.2 U/L, *P* < 0.05) and lowered the TCH (5.9 ± 1.4 vs. 5.1 ± 1.3 mmol/L, *P* < 0.05) and TG (3.8 ± 3.6 vs. 2.1 ± 1.0 mmol/L, *P* < 0.05) levels.

### 3.3. Effects of Liraglutide on Serum FGF21 Levels and Liver Fat Content

**Figure 1:**
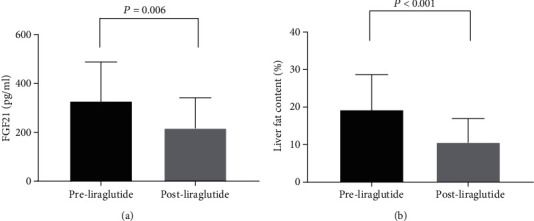
Effects of the 12-week liraglutide treatment on fibroblast growth factor (FGF) 21 levels and liver fat content. (a) FGF21 levels; (b) liver fat content.

**Figure 2:**
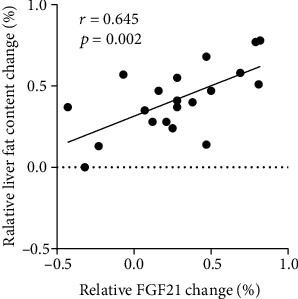
Association between relative change (%) of liver fat content and relative change (%) of FGF21 after the 12-week liraglutide treatment.

**Figure 3:**
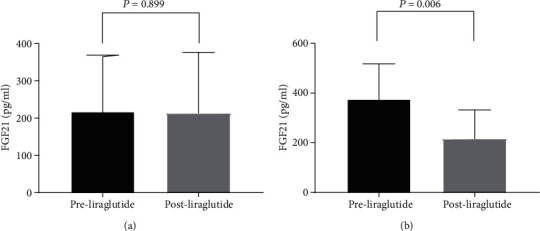
Comparison of FGF21 changes between patients with or without a significant relative decrease of liver fat content. (a) Patients without a significant relative decrease liver fat content (<29%); (b) patients with a significant relative decrease liver fat content (≥29%).

As shown in Figure 1, liraglutide treatment significantly decreased serum FGF21 levels (159.6 ± 35.7 vs. 124.2 ± 27.8 pg/ml, *P* = 0.006) (Figure 1(a) and Table 3) and liver fat content in the T2DM patients (19.3 ± 9.4 vs. 10.6 ± 6.3%, *P* < 0.001) (Figure 1(b) and Table 3).  Figure 2 illustrates a positive association between relative change of liver fat content and relative change of FGF21 after the 12-week liraglutide treatment (*r* = 0.645, *P* = 0.002). We analyzed alterations of serum FGF21 levels in patients with various relative decreases of liver fat content in the liraglutide-treated group. As shown in Figure 3, the serum FGF21 levels in patients who had a significant relative decrease in liver fat content (<29%) were not altered (95% CI: -60.0, 54.1, *P* = 0.899, Figure 3(a)); however, a significant reduction in serum FGF21 levels (95% CI: -262.8, -55.1, *P* = 0.006, Figure 3(b)) after liraglutide treatment was observed in patients who had a significant relative decrease in liver fat content (≥29%).

## 4. Discussion

In the present study, we observed that both serum FGF21 levels and liver fat content in newly diagnosed overweight patients with T2DM and NAFLD were higher than those in the controls. The results also demonstrated that liraglutide treatment could decrease liver fat content and alter plasma FGF21 levels in newly diagnosed overweight patients with T2DM. In addition, we found that liraglutide treatment improved glycemic control in patients with overweight T2DM and NAFLD, as evidenced by significant reductions in FBG (3.3 mmol/L), 2 hPBG (8.8 mmol/L), and HbA1c (3.8%).

Weight loss has been proven to be a highly effective treatment for both hepatic steatosis and fibrosis. An approximate 3%-5% reduction in body weight can attenuate steatosis [18]. In our study, we found that liraglutide treatment resulted in prominent body weight loss (4.9 kg, 6.8%) and decreased the relative liver fat content by 41.7%. Cantero et al. reported that liver fat content is related to hepatic injury biomarkers including ALT and AST [19]. Here, we found that ALT and AST levels were reduced, which may be due to the decrease in liver fat content. In accordance with our findings, Shao et al. reported that exenatide treatment for 12 weeks decreased body weight and liver fat in obese patients with T2DM and NAFLD [20]. It was also shown that a 26-week treatment with exenatide reduces liver fat content by 24% [21]. Our findings are also consistent with Petit et al., who reported that the 6-month treatment with liraglutide resulted in remarkable decreases in body weight (3.6 kg), HbA1c (2.5%), and liver fat content (31%) [22].

The study by Shao et al. used ultrasonography to evaluate liver fat, which is characterized by limited sensitivity. So we employed MRI to diagnose NAFLD. Interventions in the aforementioned studies included GLP-1 RAs and insulin or oral hypoglycemic drugs, which might bring about potential drug interactions. Therefore, we chose liraglutide monotherapy for newly diagnosed overweight T2DM patients. A previous study showed that liraglutide treatment for 12 weeks resulted in a 5 kg weight loss, which reached 6 kg when the treatment continued to 24 weeks. The decrease in blood AST levels paralleled the weight loss, indicating that weight loss might be associated with changes in hepatic injury biomarkers [3]. In contrast, Tang et al. found that the 12-week treatment with liraglutide had no effect on liver fat [23]. In contrast, the results of a randomized controlled trial revealed that the 12-week treatment with sitagliptin did not affect liver fat in NAFLD patients when compared with those taking placebo [24]. These contradictory outcomes may be due to differences in study population.

Although the protective effect of liraglutide against hepatic steatosis is highly correlated with weight loss, there are still other potential mechanisms of action of GLP-1 RAs that are independent of weight loss. Kim et al. in a recent study revealed that the combination therapy of liraglutide and FGF21 improves insulin sensitivity in the liver by increasing Akt and ERK1/2 phosphorylation [25]. Some studies suggest that GLP-1 directly reduces hepatic lipogenesis via the AMP-activated protein kinase (AMPK) and insulin signaling pathway in vitro [26, 27], which might contribute to increased hepatic fatty acid oxidation and consequent improvement of insulin sensitivity. However, other studies reported that liver does not express GLP-1 receptor (GLP-1R). Panjwani and colleagues have showed that previous observations showing the detection of GLP-1R in human and mouse liver are likely due to the lack of reliable antibody [28, 29]. Liu et al. found that in HFD-fed mice, in vivo liraglutide treatment increased hepatic FGF21 expression, but in mouse primary hepatocytes, direct liraglutide treatment generated no such stimulation. Furthermore, RNA-seq and other techniques cannot detect GLP-1R expression in the liver, and in GLP-1R knockout mice, in vivo stimulation effect of liraglutide was gone. Thus, liraglutide may interact with GLP-1R expressed in an extrapancreatic organ to upregulate hepatic FGF21 expression [30]. Here, we found that HOMA-IR was decreased after a 12-week liraglutide treatment, indicating insulin sensitivity was improved.

A recent study reported that plasma FGF21 is associated with severity of nonalcoholic steatohepatitis in obese patients with T2DM. Measurement of FGF21 may be helpful for identifying disease progression [31]. In our study, serum FGF21 levels were positively correlated with FBG, 2 hPBG, FINS, HOMA-IR, HbA1c, TCH, ALT, AST, GGT, and liver fat content at baseline (*r* = 0.717, *P* < 0.001). Samson et al. demonstrated that the combination therapy of pioglitazone and exenatide not only reduced plasma FGF21 levels but also induced a greater decrease in liver fat content than pioglitazone monotherapy. Exendin-4 treatment has also been shown to reduce liver triacylglycerol content, inhibit hepatic FGF21 protein expression, and activate hepatic AMPK phosphorylation in mice, indicating an improvement of hepatic FGF21 resistance [32]. Two studies reported that liraglutide and dietary polyphenol intervention may not only regulate FGF21 production but also its sensitivity, partially through insulin sensitivity improvement, which has been demonstrated in animal models with high-fat diet-fed mouse models [30, 33]. The significant decrease of serum total FGF21 level by GLP-1 RAs therapy may contribute to the reduced liver fat content. Moreover, in our study, the relative change of liver fat content was highly correlated with a relative change in serum FGF21 levels (*r* = 0.645, *P* = 0.002). In contrast, Liu et al. demonstrated that exenatide treatment increased serum FGF21 levels in T2DM patients [34]. The varied changes of FGF21 levels before and after treatment may be related to the degree of FGF21 resistant. This study and that study by Liu et al. both observed FGF21 stimulation and FGF21 sensitivity improvement. Li et al. reported that obese/overweight and T2DM patients show elevated plasma FGF21 level, because those patients are FGF21 resistant [35]. Liraglutide treatment can upregulate FGF21 production, which will lead to improved lipid profile. Thus, FGF21 resistance will be attenuated, resulting in reduced plasma FGF21 level [33].This may partially explain the difference in outcomes.

Patel et al. reported that a 29% relative decline in liver fat on MRI-PDFF is associated with a histologic response in NASH [36]. Therefore, 29% was designated as the cutoff value in this study. We found that the serum FGF21 levels in patients without a distinct decline in relative liver fat content (<29%) were not altered (95% CI: -60.0, 54.1, *P* = 0.899). Interestingly, when the relative reduction of liver fat content was ≥29%, the serum FGF21 levels in patients were significantly decreased (95% CI: -262.8, -55.1, *P* = 0.006). Based on the above, we speculate that FGF21 may be an effective, noninvasive biomarker for assessing the effects of liraglutide treatment on hepatic fat and glucose metabolism. A recent study of Yang et al. reported that higher baseline FGF21 levels are associated with poorer glycemic responses to exenatide in patients with type 2 diabetes [37]. Badakhshi and Jin reviewed that FGF21 may be a potential biomarker for disease diagnosis and prognosis. However, FGF21 is also recognized as a “stress hormone” and varies among different populations and fluctuate tremendously with fasting and refeeding. So it is essential to unify the technique for its clinical measurement [38].

There are a few limitations in our study. First, this study was not a randomized controlled trial, which may have resulted in potential bias in the results. Well-designed randomized controlled trials are needed to confirm our results. Secondly, even though MRI is recognized as the “golden standard” for noninvasive assessment of liver TG content, the findings were not histologically verified. Lastly, we only observed short-term effects (12 weeks), and longer exposure time to liraglutide might be needed to reveal significant effects on liver fat content and FGF21 levels.

In conclusion, we have demonstrated that liraglutide treatment in overweight patients with T2DM could decrease both liver fat content and serum FGF21 levels. FGF21 may be a potential biomarker for evaluating the effects of liraglutide treatment on hepatic glucose and lipid metabolism.

## Figures and Tables

**Table 1:** Baseline characteristics of all subjects.

	Control	Pretreatment	*P*
Clinical characteristics			
*n* (male/female)	20 (16/4)	20 (17/3)	1.000
Age (years)	38.7 ± 9.8	37.4 ± 8.9	0.676
Weight (kg)	66.9 ± 5.7	69.8 ± 6.2	0.128
BMI (kg/m^2^)	27.5 ± 0.2	27.6 ± 0.5	0.188
Waist circumference (cm)	86.2 ± 3.3	91.0 ± 4.1	<0.001^∗∗^
Biochemical and metabolic parameters			
FBG (mmol/L)	4.8 ± 0.4	9.9 ± 2.8	<0.001^∗∗^
2 hPBG (mmol/L)	5.8 ± 0.6	16.5 ± 5.5	<0.001^∗∗^
FINS (*μ*U/ml)	5.3 ± 1.9	15.6 ± 7.2	<0.001^∗∗^
HOMA-IR	1.3 ± 0.4	6.7 ± 2.9	<0.001^∗∗^
HbA1c (%)	5.2 ± 0.3	10.0 ± 2.0	<0.001^∗∗^
TCH (mmol/L)	4.6 ± 0.5	5.9 ± 1.4	<0.001^∗∗^
HDL (mmol/L)	1.4 ± 0.4	0.9 ± 0.1	<0.001^∗∗^
LDL (mmol/L)	2.7 ± 0.6	3.2 ± 1.3	0.113
TG (mmol/L)	1.2 ± 0.7	3.8 ± 3.6	0.016^∗^
ALT (U/L)	29.1 ± 19.4	56.4 ± 35.4	0.004^∗^
AST (U/L)	26.2 ± 15.1	38.3 ± 18.7	0.03^∗^
ALP (U/L)	66.1 ± 18.2	77.7 ± 24.7	0.100
GGT (U/L)	25.3 ± 20.7	79.1 ± 69.1	0.002^∗^
FGF21 (pg/ml)	124.1 ± 42.9	159.6 ± 35.7	<0.001^∗∗^
Liver fat content (%)	4.5 ± 0.6	19.3 ± 9.4	<0.001^∗∗^

Abbreviations: BMI: body mass index; FBG: fasting blood glucose; 2 hPBG: 2-hour postprandial blood glucose; FINS: fasting insulin; HOMA-IR: homeostasis model assessment of insulin resistance; HbA1c: glycated hemoglobin; TC: total cholesterol; HDL: high-density lipoprotein cholesterol; TG: triglyceride; LDL: low-density lipoprotein cholesterol; ALT: alanine amino transferase; AST: aspartate aminotransferase; ALP: alkaline phosphatase; GGT: *γ*-glutamyl transferase; FGF21: fibroblast growth factor 21. ^∗^*P* < 0.05; ^∗∗^*P* < 0.01.

**Table 2:** Associations between FGF21 and various factors at baseline in all subjects.

	*r*	*P*
Age	-0.252	0.121
Weight	0.032	0.848
BMI	0.088	0.596
Waist circumference	0.232	0.156
FBG	0.575	<0.001^∗∗^
2 hPBG	0.515	0.001^∗∗^
FINS	0.808	<0.001^∗∗^
HOMA-IR	0.788	<0.001^∗∗^
HbA1c	0.857	<0.001^∗∗^
TCH	0.389	0.014^∗^
HDL	-0.383	0.016^∗^
LDL	0.136	0.408
TG	0.286	0.078
ALT	0.649	<0.001^∗∗^
AST	0.591	<0.001^∗∗^
ALP	0.189	0.250
GGT	0.537	<0.001^∗∗^
Liver fat content	0.717	<0.001^∗∗^

Abbreviations: BMI: body mass index; FBG: fasting blood glucose; 2 hPBG: 2-hour postprandial blood glucose; FINS: fasting insulin; HOMA-IR: homeostasis model assessment of insulin resistance; HbA1c: glycated hemoglobin; TC: total cholesterol; HDL: high-density lipoprotein cholesterol; TG: triglyceride; LDL: low-density lipoprotein cholesterol; ALT: alanine amino transferase; AST: aspartate aminotransferase; ALP: alkaline phosphatase; GGT: *γ*-glutamyl transferase; FGF21: fibroblast growth factor 21. ^∗^*P* < 0.05; ^∗∗^*P* < 0.01.

**Table 3:** Effects of liraglutide treatment on clinical characteristics and biochemical and metabolic parameters.

	Pretreatment	Posttreatment	Changes	*P*
Clinical characteristics				
*n* (men/women)	20 (17/3)			
Age (years)	37.4 ± 8.9			
Weight (kg)	69.8 ± 6.2	64.9 ± 4.4	-4.9 (-6.1, -3.7)	<0.001^∗∗^
BMI (kg/m^2^)	27.6 ± 0.4	25.7 ± 0.7	-1.9 (-2.3, -1.5)	<0.001^∗∗^
Waist circumference (cm)	91.0 ± 4.1	85.0 ± 2.7	-6.1 (-7.2, -4.9)	<0.001^∗∗^
Biochemical and metabolic parameters				
FBG (mmol/L)	9.9 ± 2.8	6.3 ± 1.1	-3.3 (-5.1, -2.0)	<0.001^∗∗^
2 hPBG (mmol/L)	16.5 ± 5.5	7.8 ± 1.6	-8.7 (-11.4, -5.9)	<0.001^∗∗^
FINS (*μ*U/ml)	15.6 ± 7.2	14.7 ± 6.5	-0.9 (-4.6, 2.9)	0.636
HOMA-IR	6.7 ± 2.9	4.9 ± 2.1	-1.7 (-3.2, -0.24)	0.024^∗^
HbA1c (%)	10.0 ± 2.0	6.2 ± 0.6	-3.8 (-4.7, -2.9)	<0.001^∗∗^
TCH (mmol/L)	5.9 ± 1.4	5.1 ± 1.3	-0.8 (-1.5, -0.1)	0.028^∗^
HDL (mmol/L)	0.9 ± 0.1	1.0 ± 0.2	0.1 (-0.1, 0.1)	0.172
LDL (mmol/L)	3.2 ± 1.3	3.2 ± 1.1	0.0 (-0.4, 0.5)	0.862
TG (mmol/L)	3.8 ± 3.6	2.1 ± 1.0	-1.7 (-3.3, -0.2)	0.030^∗^
ALT (U/L)	56.4 ± 35.4	32.4 ± 23.1	-24.1 (-34.4, -13.7)	<0.001^∗∗^
AST (U/L)	38.3 ± 18.7	23.0 ± 7.9	-15.3 (-23.3, -7.3)	<0.001
ALP (U/L)	77.7 ± 24.7	68.8 ± 21.6	-8.9 (-18.4, 0.5)	0.062
GGT (U/L)	79.1 ± 69.1	46.8 ± 26.2	-32.4 (-59.1, -5.6)	0.020^∗^
FGF21 (pg/ml)	159.6 ± 35.7	124.2 ± 27.8	-110.7 (-185.8, -35.6)	0.006^∗∗^
Liver fat content (%)	19.3 ± 9.4	10.6 ± 6.3	-8.7 (-11.9, -5.5)	<0.001^∗∗^

Abbreviations: BMI: body mass index; FBG: fasting blood glucose; 2 hPBG: 2-hour postprandial blood glucose; FINS: fasting insulin; HOMA-IR: homeostasis model assessment of insulin resistance; HbA1c: glycated hemoglobin; TC: total cholesterol; HDL: high-density lipoprotein cholesterol; TG: triglyceride; LDL: low-density lipoprotein cholesterol; ALT: alanine amino transferase; AST: aspartate aminotransferase; ALP: alkaline phosphatase; GGT: *γ*-glutamyl transferase; FGF21: fibroblast growth factor 21. ^∗^*P* < 0.05; ^∗∗^*P* < 0.01.

## Data Availability

The data used to support the findings of this study are available from the corresponding author upon request.
